# Takotsubo Cardiomyopathy as a Cardiovascular Manifestation of COVID-19: A Case Report and Literature Review

**DOI:** 10.7759/cureus.30005

**Published:** 2022-10-06

**Authors:** Fadila Noor, Olushola O Ogunleye, Oluwafemi Ajibola, Shuja Malik, Valerie Cluzet

**Affiliations:** 1 Internal Medicine, Vassar Brothers Medical Center, Poughkeepsie, USA; 2 Infectious Diseases, Vassar Brothers Medical Center, Poughkeepsie, USA

**Keywords:** broken-heart syndrome, covid-19 and cardiomyopathy, covid and cardiovascular complications, takotsubo cardioyopathy, coronavirus disease (covid-19)

## Abstract

Coronavirus disease 2019 (COVID-19) has a wide range of clinical manifestations, affecting multiple organ systems. Cardiovascular manifestations of COVID-19 that have been reported include arrhythmias, myocarditis, and an increased predisposition to acute myocardial infarction. Takotsubo cardiomyopathy (TCM), which is characterized by apical ballooning of the heart leading to acute left ventricular dysfunction, is scarcely seen in COVID-19 patients. We present a case of COVID-19-associated TCM in a 68-year-old man.

A 68-year-old man with no significant past medical history presented with sudden-onset midsternal pressure-like chest pain at rest, associated with diaphoresis and shortness of breath. This occurred ten days after diagnosis of COVID-19 with mild symptoms, with no other recent physical or emotional stressors. At presentation, he was afebrile (98.5 °F), hypertensive (177/108 mmHg), tachycardic (HR 118 bpm), and saturating 100% on room air. Labs were significant for leukocytosis with 15.1 × 103 WBCs/mcL, elevated creatinine (1.46 g/dL), brain natriuretic peptide (BNP) of 156, troponin of 4 ng/mL that peaked at 16.28 ng/mL. The rapid COVID-19 test was positive. EKG showed anterolateral ST elevation and QTc interval of 446 ms. Echo showed severe hypokinesis of mid and apical segments and severely decreased left ventricular ejection fraction (LVEF)of <30%. Emergent left heart catheterization showed 75% mid left anterior descending coronary artery (LAD) stenosis and moderate right coronary artery (RCA) disease, while the ventriculogram showed a left ventricular ejection fraction of 35% with anteroapical and inferoapical akinesia suggestive of Takotsubo cardiomyopathy. The patient was placed on aspirin, ticagrelor, and atorvastatin, carvedilol, and lisinopril. EKG the next day showed a prolonged QTc of 526 ms with T-wave inversion and no ST elevations. The patient had no findings consistent with myocarditis or pheochromocytoma. He was discharged two days later. Within the next few weeks, his symptoms improved, and a follow-up echo confirmed normalization of left ventricular function.

There has been an increased incidence of Takotsubo cardiomyopathy during the COVID-19 pandemic compared to the pre-pandemic period. There is only a slight female preponderance in COVID-19-induced TCM, possibly because males are predominantly affected by COVID-19. Our patient satisfied all four Mayo Clinic criteria required for the diagnosis of TCM. Pathophysiology of TCM in COVID-19 is linked with cytokine storm and consequent catecholamine surge. Most patients improve within succeeding weeks or months. Nonetheless, the case fatality rate is high 36.5%, which is significantly higher compared to TCM patients without COVID-19.

COVID-19 has a multisystem involvement with various clinical presentations. New cardiomyopathy in COVID-19 patients should raise suspicion among clinicians regarding stress-induced cardiomyopathy.

## Introduction

Infection with severe acute respiratory syndrome coronavirus 2 (SARS-CoV-2) virus, which causes coronavirus disease 2019 (COVID-19), was first reported in Wuhan, the capital of Hubei, China in December 2019, and since then it has spread worldwide. COVID-19 has a wide array of clinical presentations, ranging from asymptomatic to mild respiratory illness to respiratory failure, shock, and death [1]. The presentation is not limited to the respiratory system; rather, it affects multiple organ systems, including the cardiovascular system. Cardiovascular sequelae include cardiomyopathy, myocarditis, arrhythmia, acute myocardial infarction, and venous thromboembolism [1]. Takotsubo cardiomyopathy (TCM), also called broken heart syndrome or apical ballooning syndrome, is a syndrome of reversible wall motion abnormality that results in left ventricular dysfunction. The apical ballooning is usually triggered by physical or emotional stressors and manifests as acute left ventricular dysfunction. The proinflammatory cytokine storm and procoagulant state in COVID-19 increases the risk of cardiac injury, stunning, coronary vasospasm, and acute myocardial infarction [2]. We report a case of TCM occurring as a cardiovascular manifestation of mild COVID-19.

## Case presentation

A 68-year-old man with no significant past medical history and unvaccinated against the SARS-CoV-2 virus tested positive for COVID-19 ten days prior to presentation in the setting of mild fatigue, nonproductive cough, chills, and diarrhea. The patient presented to the hospital with sudden onset of mid-sternal pressure-like chest discomfort. The chest discomfort was associated with diaphoresis and shortness of breath. He denied exposure to stressors except for the recent COVID-19 infection. He had no smoking history and no personal or family history of coronary artery disease.

**Figure 1:**
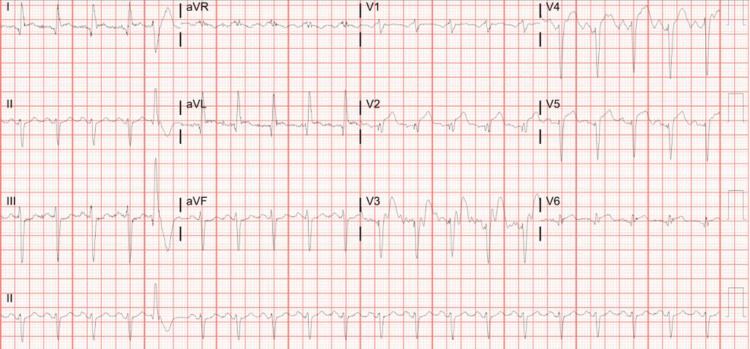
EKG at presentation, showing ST elevations in leads V2-V5

On presentation, the patient was afebrile (98.5 °F), hypertensive (177/108 mmHg), tachycardic (118 bpm), and saturating 100% breathing ambient air at 20 breaths/min. Laboratory evaluation (Table 1) was significant for leukocytosis of 15.1 x 109 WBC/L with neutrophilic predominance of 80.9%, creatinine of 1.46 mg/dL, mild hyponatremia of 132 mmol/L, elevated brain natriuretic peptide (BNP)of 156 pg/mL, slightly elevated aspartate Transaminase (AST)of 64 IU/L, and elevated troponin-I level of 4 ng/mL. The rapid antigen test for COVID-19 was positive. EKG showed sinus tachycardia with occasional premature ventricular complexes with ST elevation pronounced in the anterolateral leads and QTc interval of 446 ms (Figure 1).

**Table 1:** Laboratory findings during hospital stay WBC: White Blood Cell; INR: International normalized ratio; PT: Prothrombin time; APTT: Partial Thromboplastin Time; BUN: Blood Urea Nitrogen; CO2 (Carbon dioxide in blood); ALT: Alanine Transaminase; AST: Aspartate Transaminase; ALP: Alkaline Phosphatase; BNP: Brain Natriuretic Peptide; HDL: High-density lipoprotein; LDL: Low-density lipoprotein; CRP: C-reactive protein; TSH: Thyroid Stimulating Hormone; gm/dL: grams per deciliter; mg/dL: milligrams per deciliter; ng/mL: nanograms per milliliter; mmol/L: millimoles per liter; IU/L: international units per liter; gm/L: grams per Liter; pg/mL: picograms per milliliter; mcIU/mL: micro-international units per milliliter

Labs	Initial Lab Finding	5 hours later	10 hours later	Normal
WBC	15.1 x 10 (9)/L (elevated); 80.9% neutrophil; 12% lymphocyte			3.5-10 (9)/L; Neutrophil:40-75%; Lymphocyte: 20-45%
Hemoglobin	14.2 g/dL (normal)			13.5-17 g/dL
Platelet	353 x 10 (9)/L (normal)			150-400 x 10(9)/L
INR	1.1 ratio (normal)			0.9-1.2 ratio
PT	12.9 seconds (normal)			10.2-12.9 seconds
APTT	31.4 seconds (normal)			27.4-32.8 seconds
D-dimer	375 ng/mL (normal)			0-230 ng/mL
Glucose	150 mg/dL (elevated)			65-99 mg/dL
BUN	15.9 mg/dL (normal)			6-20 mg/dL
Creatinine	1.46 mg/dL (elevated)			0.7-1.2 mg/dL
Sodium	132 mmol/L (low)			136-145 mmol/L
Potassium	3.8 mmol/L (normal)			3.5-5.1 mmol/L
Chloride	100 mmol/L (normal)			98-107mmol/L
CO2	18 mmol/L (low)			22-29 mmol/L
Calcium	9.1 mg/dL (normal)			8.6-10 mg/dL
Phosphorus	2.6 mg/dL (normal)			2.6-4.7 mg/dL
ALT	20 IU/L (normal)			7-40 IU/L
AST	64 IU/L (elevated)			15-41 IU/L
ALP	88 IU/L (normal)			38-126 IU/L
Albumin	3.9 gm/dL (normal)			3.5-5 gm/L
Globulin	3.6 gm/dL (normal)			2-4.5 gm/dL
BNP	156 pg/mL (elevated)			<100 pg/mL
Magnesium	1.8 mg/dL (normal)			1.6-2.6 mg/dL
Cholesterol	208 mg/dL (elevated)			<199 mg/dL
Triglyceride	93 mg/dL (normal)			72-175 mg/dL
HDL cholesterol	43 mg/dL (normal)			>40 mg/dL
LDL	146 mg/dL (elevated)			10-100 mg/dL
CRP	8.6 mg/dL (elevated)			<1 mg/dL
Hemoglobin A1C	6% (Pre-diabetes)			<5.6%
TSH	3.27 mclU/mL (Normal)			0.34-5.6 mcIU/mL
Troponin-I	4.00 ng/mL (elevated)	8.87 ng/mL (elevated)	16.28 ng/mL (elevated)	<0.09 ng/mL

**Figure 2:**
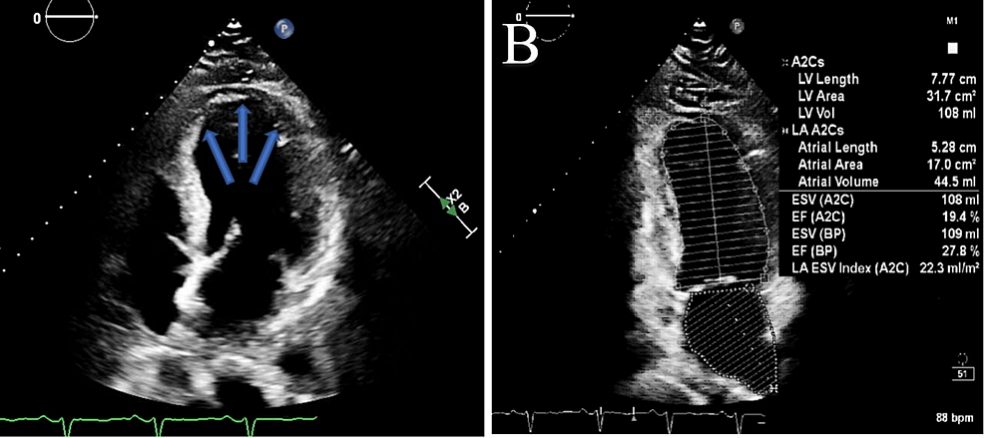
A and B: Echocardiogram illustrating the apical ballooning of the heart (blue arrows in A), with severely decreased left ventricular systolic function (left ventricular ejection fraction <30%) and severe hypokinesis of mid and apical segments, suggestive of stress-mediated cardiomyopathy

**Figure 3:**
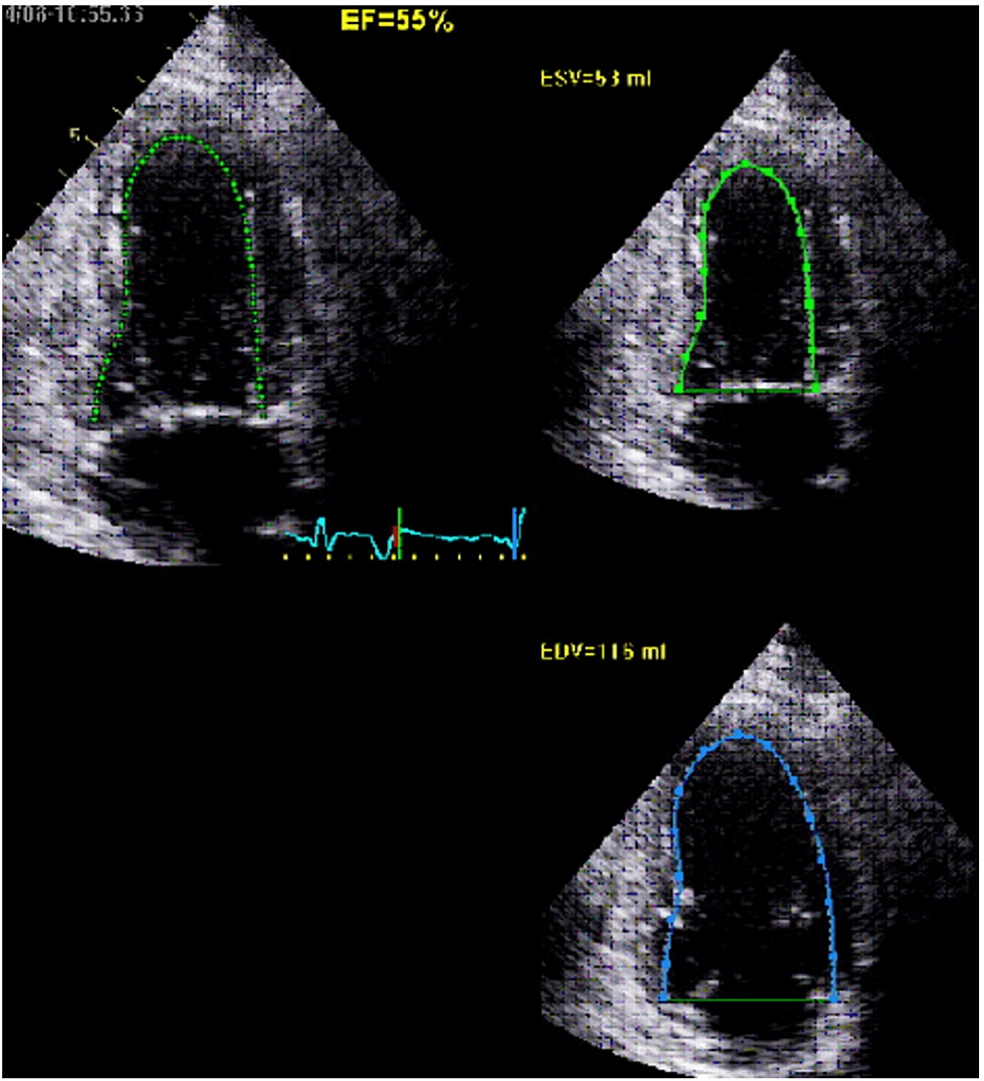
Three-month echocardiogram showing normal left ventricular chamber size, mildly decreased left ventricular ejection fraction estimated at 40-49%, with akinetic apex, and hypokinetic inferior wall and anteroseptal wall

Given concern for acute coronary syndrome, the patient was started on aspirin, ticagrelor, heparin, carvedilol, and nitroglycerin. Five hours later, the repeat troponin-I level increased to 8.87 ng/mL. The patient underwent emergent left heart catheterization that showed 75% mid-left anterior descending coronary artery (LAD) stenosis and moderate right coronary artery (RCA) disease that was not consistent with ST-elevation myocardial infarction (STEMI). Ventriculography showed anteroapical and inferoapical akinesia consistent with TCM. Echocardiogram confirmed severe hypokinesis of the mid and apical segments of the left ventricle, suggestive of TCM, with severely reduced left ventricular ejection fraction (LVEF) < 30% (Figures 2A, 2B). The troponin-I level peaked at 16.28 ng/mL. By hospital day two, the patient’s chest discomfort had resolved, his EKG had normalized, his troponin-I levels were trending downwards, and he was hemodynamically stable; hence, the patient was discharged home on aspirin, clopidogrel, atorvastatin, and lisinopril with scheduled outpatient cardiology follow up. The outpatient three-month follow-up echocardiogram showed improvement in LVEF to 40-49% (Figure 3).

## Discussion

An increased incidence of TCM has been reported during the COVID-19 pandemic compared to the pre-pandemic period. In a retrospective cohort study at a cardiac catheterization lab in Northeast Ohio, TCM was reported to have an incidence 4.58 times higher during the COVID-19 pandemic compared to the pre-pandemic period [3]. With COVID-19, there is a markedly high level of cortisol, along with high catecholamine levels, which could result in sympathetic overstimulation and exert a direct effect on cardiomyocytes, thereby leading to the development of TCM [4]. The proinflammatory cytokine storm and procoagulant state in COVID-19 increases the risk of cardiac injury, stunning, coronary vasospasm, and acute myocardial infarction [2]. Factors contributing to this recently increased TCM incidence include COVID-19-induced TCM as well as other psychological, social, and economic stressors associated with the COVID-19 pandemic [3]. Specifically, a case of TCM triggered by “COVID-19 pandemic anxiety” has been reported [5]. Unlike typical cases of TCM, which have a 9:1 female preponderance, in COVID-19-induced TCM, there is only a slight female preponderance (55.5%-59.6%), possibly because males are predominantly affected by COVID-19 [6,7,8].

The Mayo clinic criteria are used for the diagnosis of TCM - all four criteria must be present for the diagnosis of TCM [1,9]. They include [9]: (1) transient LV hypokinesis, akinesis, or dyskinesis in the mid-segments with or without apical involvement, with extension of the regional wall motion abnormalities beyond the distribution of a single epicardial coronary vessel, often (but not always) triggered by a stressor; (2) absence of angiographic evidence of acute plaque rupture or obstructive coronary artery disease; (3) new abnormalities on electrocardiography (typically ST elevation and/or T-wave inversion) or modest cardiac troponin elevation; and (4) absence of concomitant myocarditis or pheochromocytoma.

Although left heart catheterization showed 75% mid-LAD stenosis and moderate RCA disease, this was not consistent with the EKG changes that suggested ST-elevation myocardial infarction which would require total occlusion. Also, the ventriculography showed anteroapical and inferoapical akinesia, while echocardiography confirmed severe hypokinesis of mid and apical segments. These findings cannot fully be attributed to the LAD area of distribution or explained by the moderate RCA disease and hence are more consistent with TCM. 

A variety of emotional and physical triggers for TCM are reported, including receiving bad news, a sudden surprise, and excessive physical stress (causes as varied as domestic violence, severe pain, acute illness, or intense surgical/medical procedures) [10]. Other than our patient’s recent diagnosis of COVID-19, no other stressor was identified as the trigger for TCM. COVID-19 is known to increase physical and psychosocial stressors in patients [11]. The pathophysiology of TCM in COVID-19 has been linked with the cytokine storm and consequent catecholamine surge [7,12]. Increased levels of inflammatory markers such as C-reactive protein and procalcitonin are usually seen during periods of stress and/or myocardial injury, as are myoglobin, creatine kinase, and N-terminal pro B-type natriuretic peptide [7,12]. Studies have shown that COVID-19 patients, compared to matched controls, have lower LVEF, higher left ventricle (LV) volume, higher levels of troponin, and higher cardiac MRI markers of inflammation [7, 13]. In a review of 27 cases of COVID-19-associated TCM, the most reported EKG changes included nonspecific ST or T-wave changes, diffuse PR interval and QTc prolongation; and echocardiography showed apical ballooning in most cases [7]. Another systematic review of 52 cases of COVID-19-associated TCM identified a median LVEF of 30% [8].

There are no specific guidelines targeting the management of COVID-19-induced TCM; rather, management of COVID-19-induced TCM is similar to non-COVID-19-induced TCM. The current treatment for TCM involves close monitoring and supportive care during the acute phase [14]. Mild cases of COVID-19-induced TCM without hypotension or hemodynamic instability are usually managed with medications typically used for heart failure with reduced ejection fraction, which include venodilators (nitrates), diuretics (loop diuretics, aldosterone antagonists), arterial vasodilators (angiotensin-converting enzyme inhibitors, angiotensin II receptor blockers, neprilysin inhibitor, hydralazine) and beta blockers [14, 15]. Beta blockers are usually avoided during the acute phase due to the risk of cardiogenic shock. In the cases of TCM complicated by hypotension and cardiogenic shock without left ventricular outflow tract obstruction, the management includes intravenous fluid, inotropes (dobutamine, milrinone) and mechanical circulatory support [14, 15]. When left ventricular outflow tract obstruction occurs in addition to hypotension and cardiogenic shock in cases of TCM, positive inotropes are generally avoided due to the risk of worsening the obstruction [14, 15]; the management of these patients includes intravenous fluids, beta blockers, alpha-1 receptor agonists, and mechanical circulatory support in refractory cases [14, 15, 16]. A review of a consortium of 26 centers involving 1,750 patients with TCM showed the presence of ventricular thrombus in 1.3% of these patients. Anticoagulation is typically unnecessary in cases of TCM, but in TCM patients with left ventricular thrombus and severe LV dysfunction, anticoagulation is needed to prevent embolism [14, 17].

Most patients with COVID-19-induced TCM show improvement within weeks or months, as assessed either via clinical findings or on repeat echocardiography that shows improvement in LV function [7]. Nonetheless, the case fatality rate in COVID-19-induced TCM is as high as 36.5%. This is significantly higher compared to TCM patients without COVID-19, which ranges from 0.95-2.3% in those without cardiogenic shock to 13.6-23.5% in those with cardiogenic shock [8]. Similarly, TCM in COVID-19 patients seems to be associated with more adverse outcomes. Compared to those without TCM, cases of COVID-19 complicated by TCM typically have a more critical illness [8]. The prognostic factors in patients with TCM include the patient’s age and the type of triggering event. Older age and the presence of physical triggers like COVID-19 are associated with worse outcomes and higher mortality in relation to the degree of myocardial involvement [7].

## Conclusions

COVID-19 has multisystemic involvement with various clinical presentations. This case highlights TCM as one of the effects of COVID-19 on the cardiovascular system. Patients with recent COVID-19 infection presenting with chest discomfort and ST or T-wave changes (whether specific or nonspecific) or QTc prolongation on EKG should be evaluated for TCM.
